# Intra-abdominal pressure alterations after large pancreatic pseudocyst transcutaneous drainage

**DOI:** 10.1186/1471-230X-9-42

**Published:** 2009-06-06

**Authors:** Theodossis S Papavramidis, Vassilis Duros, Antonis Michalopoulos, Vassilis N Papadopoulos, Daniel Paramythiotis, Nick Harlaftis

**Affiliations:** 1 1st Propedeutic Department of Surgery, AHEPA University Hospital, Aristotle's University of Thessaloniki, Macedonia, Greece

## Abstract

**Background:**

Acute pancreatitis leads to abdominal hypertension and compartment syndrome. Weeks after the episodes pancreatic fluids sometimes organize to pseudocysts, fluid collections by or in the gland.

Aims of the present study were to evaluate the intra-abdominal pressure (IAP) induced by large pancreatic pseudocysts and to examine the effect of their transcutaneous drainage on IAP.

**Methods:**

Twenty seven patients with a pancreatic pseudocyst were included. Nine patients with pseudocysts greater than 1l (group A) had CT drainage and eighteen (volume less than 1l) were the control group. The measurements of group A were taken 6 hours before and every morning after the drainage, while for group B, two measurements were performed, one at the day of the initial CT and one 7 days after. Abdominal compliance (Cabd) was calculated. Data were analyzed using student's *t*-test.

**Results:**

Baseline IAP for group A was 9.3 mmHg (S.D. 1.7 mmHg), while the first post-drainage day (PDD) IAP was 5.1 mmHg (S.D. 0.7 mmHg). The second PDD IAP was 5.6 mmHg (S.D. 0.8 mmHg), the third 6.4 mmH (S.D. 1.2 mmHg)g, the fourth 6.9 mmHg (S.D. 1.6 mmHg), the fifth 7.9 mmHg (S.D. 1.5 mmHg), the sixth 8.2 mmHg (S.D. 1.4 mmHg), and the seventh 8.2 mmHg (S.D. 1.5 mmHg). Group B had baseline IAP 8.0 mmHg (S.D. 1.2 mmHg) and final 8.2 mmHg (S.D. 1.4 mmHg). Cabd after drainage was 185.6 ml/mmHg (SD 47.5 ml/mmHg).

IAP values were reduced between the baseline and all the post-drainage measurements in group A. IAPs seem to stabilize after the 5^th ^post-drainage day. Baseline IAP was higher in group A than in group B, while the two values, at day 7, were equivalent.

**Conclusion:**

The drainage of large pancreatic pseudocyst reduces IAP. Moreover, the IAP seems to rise shortly after the drainage again, but in a way that it remains inferior to the initial value. More chronic changes to the IAP are related to abdominal cavity's properties and have to be further studied.

## Background

Intra-abdominal pressure (IAP) is the pressure maintained in the abdominal cavity. It can be measured using various techniques, but the most used and the easiest to apply is the transvesical [1-3]. Under normal conditions – no pathologies – and with the patient calm and at supine position, IAP ranges from 1 to 6.5 mmHg [2-4].

It is well known that acute pancreatitis is one of the most frequent reasons leading to abdominal hypertension (IAH) and abdominal compartment syndrome (ACS) [5-8]. The management of IAH and ACS has many aspects [9-12] concerning the decompression of the abdomen and the amelioration of the condition of the patient. After the acute episode of pancreatitis, free pancreatic fluids sometimes organize weeks after the episodes, to pseudocysts. Pancreatic pseudocysts are pancreatic fluid collections by or in the gland. The size of the pseudocysts varies from small (<2 cm), to medium (2–6 cm), and large (>6 cm), mirroring the amount of fluid contained in the cyst.

The present study was based on the hypothesis that large pancreatic pseudocysts tend to alter IAP. Aims of the present study were to evaluate alterations of IAP due to large pancreatic pseudocysts and to examine the effect of transcutaneous pseudocyst drainage on IAP.

## Methods

The present prospective study lasted from 1 Sep 2006 to 31 Dec 2007. Twenty seven patients with a pancreatic pseudocyst, treated in the 1^st ^Propedeutic Department of Surgery of AHEPA University hospital in Thessaloniki were included. The study was approved by the ethics committee of AHEPA University Hospital and written informed consent was obtained by all patients.

Two groups were formed. All patients that fulfilled the below-mentioned criteria entered group A. The criteria were: (i) presence of large pseudocyst (volume of pseudocyst calculated greater than 1l), (ii) no signs of chronic pancreatitis (e.g pain or CT findings), (iii) no signs of acute pancreatitis (e.g pain, amylasemia, CT findings) present for the last 2 months. In group A 9 patients (6 males and 3 females) were included. On the other hand, group B included the remaining 18 patients (10 males, 8 females). The mean age of the patients was 72.2 years (range 64 to 78 years). The Body Mass Index averaged 27.2 kg/m^2 ^(S.D. 3.2 kg/m^2^) for group A and 26.8 kg/m^2 ^(S.D. 2.7 kg/m^2^) for group B. The mean volume of the initially drained pseudocyst fluid was 2313 ml (ranging from 1200 to 3200 ml, S.D 825 ml). The mean volume of the non-drained pseudocysts (Group B) was calculated as 353 ml (ranging from 240 to 540 ml, S.D. 97 ml). The demographic data for both groups is showed in table 1.

**Table 1:** Patients Demographics

	Group A	Group B	p
**Age (y)**	71.9	72.5	ns
**Gender**			
**(Male/Female)**	6/3	10/8	
**Mean BMI (kg/m2)**	27.2	26.8	ns
**Mean Fluid Volume (ml)**	2313	353	0.001
**Baseline IAP (mmHg)**	9.3	8.0	0.001

In order to measure IAP in group A, a Foley catheter was inserted into the urinary bladder using a standard sterile technique 6 hours before the Computed Tomography (CT) guided transcutaneous drainage of the pseudocyst. The bladder was filled with 50 mL of sterile saline using a closed-system technique [13]. The hydrostatic pressure in the bladder was obtained by connecting the catheter to a pressure transducer through sterile tubing [14]. Pressure measurements were made in cmH_2_O and then converted into mmHg. The zero reference point for the measurements was set at the symphyse pubis. The median value of three separate measurements was recorded with the patient relaxed in the supine position. The measurements were taken 6 hours before the drainage and at 8 o'clock every morning, for the 7 days following the drainage. Concerning group B, two measurements were performed, one at the day of the initial CT and one 7 days after. On the 7^th ^day a control CT was performed in order to evaluate the condition of the abdominal cavity. In group A, no remaining pseudocyst was detected.

Abdominal wall compliance (Cabd) was calculated using the formula: Cabd = ΔIntra-abdominal Volume (ΔIAV)/ΔIAP [15]. The above formula was transformed to Cabd = -Volume of fluid drained by the pseudocyst/Post drainage IAP – Baseline IAP.

Data was analyzed using standard statistical methods. Descriptive statistics including means, ranges, and standard deviations were used to describe the IAP measurement for each subject. Comparison between participants were made using a paired Student's *t*-test after performing one sample Kolmogorov-Smirnof test which proved that distribution was normal in all three time instances. A *p *value of 0.05 was considered significant for all tests.

## Results

Concerning group A, the mean baseline IAP (D0) was 9.3 mmHg (range, 6.6–12.5 mmHg, S.D. 1.7 mmHg). On the same day (D0), group B presented IAP equal to 8.0 mmHg (range, 6.6–9.2 mmHg, S.D 1.2 mmHg) [p = 0.001].

The final IAP (D7) for group A was 8.2 mmHg (range 6.6–11.4 mmHg, S.D 1.5 mmHg), while for group B it was 8.2 mmHg (range 6.6–8.8 mmHg, S.D 1.4 mmHg) [p = ns].

Mean Cabd, for group A, was 185.6 ml/mmHg (range from 120.6 to 242.2 ml/mmHg, SD 47.5 ml/mmHg).

**Figure 1:**
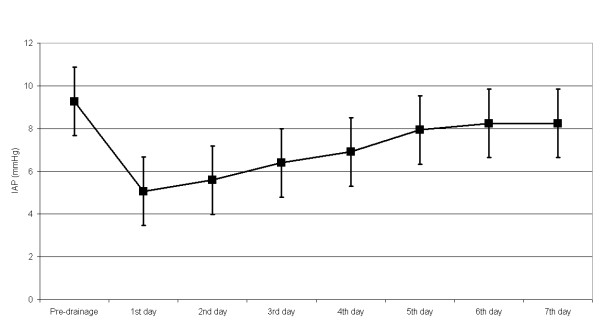
**IAP variation before and after CT-guided pseudocyst drainage**.

Finally, the evolution of the IAPs in group A, after the drainage is displayed in Figure 1.

Concerning group A, paired student's T-test showed statistically significant IAP differences between the baseline and all the post-drainage values. Furthermore, it is important to notice that IAPs seem to stabilize after the 5^th ^post-drainage day since no statistical difference appears between 5^th^, 6^th ^an 7^th ^PDD.

## Discussion

In the present study the baseline IAP values were significantly elevated in group A in comparison with group B patients. The fact that pancreatic pseudocysts are positioned retroperitoneally doesn't alter the fact that they occupy abdominal space and therefore increase IAP. This seems very logical since the volume of the fluid contained in the large pseudocysts of group A occupies a greater percentage of the total abdominal volume than that of the small pseudocysts of group B patients. These chronically increased IAPs are below pressures inducing IAH (≥ 12 mmHg), as defined by world society of the abdominal compartment syndrome (WSACS) [16-18]. By definition, those IAPs cause no clinically appreciable alterations, but special attention has to be paid when additional pathologies are present. In order to prevent the accumulation of multiple pathologies and the off-balancing of IAP towards IAH and ACS, we believe that large pancreatic pseudocyst drainage has to be done as early as possible.

The immediate post-drainage IAP values on the 1^st ^day were statistically significantly lower in comparison to the pre-drainage ones. This appears to be easily explained since the decompression of the abdominal cavity was achieved by removing the fluid of the pseudocyst. Simple laws of physics suffice to describe the alteration of IAP immediately after the drainage. The Pascal principle implies that pressure equals force divided by the surface. Given the fact that the surface remains stable, after the drainage, the force is the sole factor influencing IAP. The force implied is proportional to the volume of the intra-abdominal structures. We can easily understand that when the intra-abdominal volume decreases then the force is reduced and therefore the IAP decreases. As a conclusion, the immediate decrease of the IAP is due to physical characteristics of the abdominal cavity.

IAPs observed during the first five days after the drainage were statistically lower than these observed in group B and on days 6 and 7 of group A patients. Furthermore, there is a gradual increase of the IAP starting on day 1 and reaching a plateau on day 5. This gradual increase of the IAP seems to have a multi-variant component, since IAP is maintained by several factors such as: (i) characteristics of the abdominal wall – anterior and posterior-, (ii) properties of the diaphragm, (iii) volume of the organs in the abdomen, (iv) intra-thoracic pressure, (v) pain and (vi) muscle contraction. The above mentioned factors seem to adapt gradually to the new conditions leading to a re-escalation to the new IAP that stabilizes after the 5^th ^post-drainage day.

For the present study 50 ml of saline water were instilled ml into the bladder in order to perform the IAP measurement. However, WSACS advocated instilling a maximal amount of 25 ml into the bladder [16]. Unfortunately the study protocol had already started when the guidelines were published. Despite the fact that recent data showed that instilling 50 ml may increase intrinsic bladder pressure [19-22], we believe that the results of this study were not affected, because the aim was not to simply measure the IAP, but to evaluate differences of baseline and post- drainage IAP.

The clinical significance of the elevated IAP is not clear. Elevated IAP due to pseudocysts probably has for the moment only pure scientific value. However, the fact that draining this pseudocyst reduces IAP may help in confronting with problems induced by the pseudocyst even in IAP value below IAH when multiple pathologies are accumulated.

## Conclusion

The present study demonstrates that the drainage of large pancreatic pseudocyst reduces the IAP both in an acute and in a chronic basis. Moreover, the IAP seems to rise shortly after the drainage again, but it remains at all time inferior to the initial value. More chronic changes to the IAP are related to abdominal cavity's properties which need more time to alter, and have to be further studied.

## Competing interests

The authors declare that they have no competing interests.

## Authors' contributions

PTS Data retrieving and study designing, involved in drafting the manuscript and revising it critically for important intellectual content. DV Data retrieving and study designing, involved in drafting the manuscript and revising it critically for important intellectual content. MA Data retrieving, involved in revising the draft critically for important intellectual content. PD Data analysis and drainage of the cysts, involved in revising the draft critically for important intellectual content. PVN Data analysis and drainage of the cysts, involved in revising the draft critically for important intellectual content. HN Strategic planning for the treatment of the patients, involved in revising the draft critically for important intellectual content. All authors have read and approved the final manuscript

## Pre-publication history

The pre-publication history for this paper can be accessed here:

http://www.biomedcentral.com/1471-230X/9/42/prepub
